# Supplementary material for: AutoXAI4Omics: an automated explainable AI tool for omics and tabular data
Source: Brief Bioinform. 2024 Nov 22;26(1):bbae593. doi: 10.1093/bib/bbae593 (PMC11583442; doi:10.1093/bib/bbae593)
Supplement: Supplementary_information_togo_reviewed_bbae593 [file supplementary_information_togo_reviewed_bbae593.docx]

**Title: AutoXAI4Omics: an Automated Explainable AI tool for Omics and tabular data**

**Authors:** James Strudwick^a,1^, Laura-Jayne Gardiner^a,1^, Kate Denning-James^b^, Niina Haiminen^c^, Ashley Evans^a^, Jennifer Kelly^a^, Matthew Madgwick^a^, Filippo Utro^c^, Ed Seabolt^d^, Christopher Gibson^a^, Bharat Bedi^a^, Daniel Clayton^e^, Ciaron Howell^e^, Laxmi Parida^c^, Anna Paola Carrieri^a,∗^

*^1^Authors contributed equally to the manuscript.*

** Correspondence should be addressed to Anna Paola Carrieri*

**Supplementary Information**

Supplementary Tables S1-S3

Supplementary Figure 1

Supplementary Data files S1-S9 (formats .json, .csv)

**Supplementary Tables**

**Table S1. Output performance metrics for ML models generated from AutoXAI4Omics for example task: *Binary classification in plant genomics (Barley).*** This is the default table generated as output in folder “Results” file name: scores__performance_results_testset.csv. Train refers to the training dataset for each ML model and Test refers to the held-out test dataset used for model evaluation.

| **model** | **accuracy_score_Train** | **accuracy_score_Test** | **f1_score_Train** | **f1_score_Test** | **f1_score_PerClass_Train** | **f1_score_PerClass_Test** |
| --- | --- | --- | --- | --- | --- | --- |
| **RandomForestClassifier** | 0.9973856209150330 | 0.9791666666666670 | 0.9973856209150330 | 0.9791666666666670 | [0.99509804 0.99821747] | [0.96078431 0.9858156 ] |
| **AdaBoostClassifier** | 1.0 | 0.9791666666666670 | 1.0 | 0.9790316901408450 | [1. 1.] | [0.96       0.98591549] |
| **KNeighborsClassifier** | 0.9790849673202610 | 0.9739583333333330 | 0.9793283064360210 | 0.9740384117057670 | [0.96226415 0.98553345] | [0.95145631 0.98220641] |
| **AutoXGBoost** | 1.0 | 0.9895833333333330 | 1.0 | 0.9895158450704230 | [1. 1.] | [0.98       0.99295775] |
| **AutoLGBM** | 0.9816993464052290 | 0.9791666666666670 | 0.9816114463145270 | 0.9790316901408450 | [0.96517413 0.98758865] | [0.96       0.98591549] |
| **AutoSKLearn** | 0.9816993464052290 | 0.9635416666666670 | 0.9816993464052290 | 0.9634255938844770 | [0.96568627 0.98752228] | [0.93069307 0.97526502] |
| **AutoKeras** | 1.0 | 0.9791666666666670 | 1.0 | 0.9792925824175830 | [1. 1.] | [0.96153846 0.98571429] |

**Table S2. Output performance metrics for ML models generated from AutoXAI4Omics for example task: *Multi-class classification in human transcriptomics.*** This is the default table generated as output in folder “Results” file name: scores__performance_results_testset.csv. Train refers to the training dataset for each ML model and Test refers to the held-out test dataset used for model evaluation.

| **model** | **accuracy_score_Train** | **accuracy_score_Test** | **f1_score_Train** | **f1_score_Test** | **f1_score_PerClass_Train** | **f1_score_PerClass_Test** |
| --- | --- | --- | --- | --- | --- | --- |
| **RandomForestClassifier** | 0.9924812030075190 | 0.9705882352941180 | 0.9924826777151760 | 0.9701525054466230 | [0.99029126 1.         0.98989899] | [1.         0.93333333 0.96296296] |
| **AdaBoostClassifier** | 1.0 | 0.9411764705882350 | 1.0 | 0.9407407407407410 | [1. 1. 1.] | [0.92307692 0.93333333 0.96296296] |
| **KNeighborsClassifier** | 0.9774436090225560 | 0.9411764705882350 | 0.9774436090225560 | 0.9417086834733890 | [0.97029703 1.         0.97029703] | [0.96       0.93333333 0.92857143] |
| **AutoXGBoost** | 1.0 | 0.8823529411764710 | 1.0 | 0.8769182886829950 | [1. 1. 1.] | [0.85714286 0.76923077 0.96296296] |
| **AutoLGBM** | 1.0 | 0.9411764705882350 | 1.0 | 0.9417086834733890 | [1. 1. 1.] | [0.96       0.93333333 0.92857143] |
| **AutoSKLearn** | 0.9924812030075190 | 0.7941176470588240 | 0.9924826777151760 | 0.785171568627451 | [0.99029126 1.         0.98989899] | [0.66666667 0.93333333 0.8125    ] |
| **AutoKeras** | 0.9774436090225560 | 0.8823529411764710 | 0.9774436090225560 | 0.883461210571185 | [0.97029703 1.         0.97029703] | [0.86956522 0.93333333 0.86666667] |

**Table S3.** Feature comparison between OmicLearn and AutoXAI4Omics. In bold we highlight features that OmicLearn lacks compared to AutoXAI4Omics.

* Free deployment is available at: <https://ol-v14.streamlit.app/>

** Paid software as a service is available, contact author for more information. Software can be incorporated into any user service.

| **Feature comparison** |  | **OmicLearn** | **AutoXAI4Omics** |
| --- | --- | --- | --- |
| **Use case** | Exploration | Y | Y |
|  | **Production** | **N** | **Y** |
| **Problem types** | Binary Classification | Y | Y |
|  | **Multi-class Classification** | **N** | **Y** |
|  | **Regression** | **N** | **Y** |
| **Data** | **Data size** | **Up to 100MB** | **No limit** |
|  | Data types | Proteomics, other omics | Omics, tabular data |
| **User Interface** | **CLI** | **N** | **Y** |
|  | **Docker Image** | **N** | **Y** |
|  | Local dashboard | Y | N |
|  | SaaS | Y* | Y** |
| **Feature and samples processing** | Random seed setting | Y | Y |
|  | **Omics specific pre-processing** | **N** | **Y** |
|  | Imputation | Y | N |
|  | EDA (dim reduction & clustering) | Y | N |
|  | Standardisation | Y | Y |
|  | **Class balancing methods** | **N** | **Y** |
| **Feature Selection** | User specified number | Y | Y |
|  | **Automated selection** | **N** | **Y** |
|  | User specified method | Y | Y |
|  | Number available methods | 4 | 3 |
| **Models** | AdaBoost | Y | Y |
|  | XGBoost | Y | Y |
|  | SkLearn models | Y | Y |
|  | **AutoXGBoost** | **N** | **Y** |
|  | **Auto-LightGBM** | **N** | **Y** |
|  | **Auto-Keras** | **N** | **Y** |
|  | **Auto-sklearn** | **N** | **Y** |
| **Model handling** | Trainable models per run | 1 | as many as specified |
|  | **Hyper-tuning** | **N** | **Y** |
|  | Cross validation | Y | Y |
|  | **Best Model selection** | **N** | **Y** |
| **Explainability** | Feature importance | Y | N |
|  | **SHAP** | **N** | **Y** |
|  | **Permutation importance** | **N** | **Y** |
| **Outputs** | **Model files** | **N** | **Y** |
|  | Metric files | Y | Y |
|  | Image files | Y | Y |


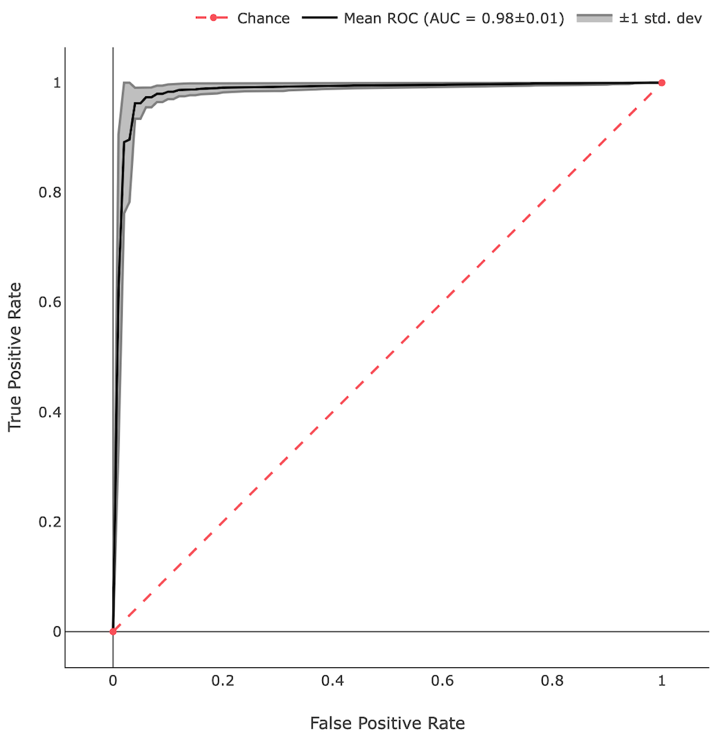


**Figure S1**. ROC curve for XGBoost as computed by OmicLearn for the binary classification use case: plant genomics.
